# A Novel Glycolysis-Related Long Noncoding RNA Signature for Predicting Overall Survival in Gastric Cancer

**DOI:** 10.3389/pore.2022.1610643

**Published:** 2022-11-07

**Authors:** Jianmin Zeng, Man Li, Kefan Dai, Bingyu Zuo, Jianhui Guo, Lu Zang

**Affiliations:** ^1^ Department of General Surgery, Ruijin Hospital, Shanghai Jiao Tong University School of Medicine, Shanghai, China; ^2^ The Affiliated Hospital of Kunming University of Science and Technology, The First People’s Hospital of Yunnan Province, Kunming, China; ^3^ The First Affiliated Hospital of Bengbu Medical College, Bengbu, China; ^4^ Second Department of General Surgery, The First People’s Hospital of Yunnan Province, The Affiliated Hospital of Kunming University of Science and Technology, Kunming, China

**Keywords:** lncRNA, gastric cancer, TCGA, immune infiltration, prognostic signature

## Abstract

**Background:** The aim of this study was to construct a glycolysis-related long noncoding RNA (lncRNA) signature to predict the prognosis of patients with gastric cancer (GC).

**Methods:** Glycolysis-related genes were obtained from the Molecular Signatures Database (MSigDB), lncRNA expression profiles and clinical data of GC patients were obtained from The Cancer Genome Atlas database (TCGA). Furthermore, univariate Cox regression analysis, Least Absolute Shrinkage and Selection Operator (LASSO) and multivariate Cox regression analysis were used to construct prognostic glycolysis-related lncRNA signature. The specificity and sensitivity of the signature was verified by receiver operating characteristic (ROC) curves. We constructed a nomogram to predict the 1-year, 3-year, and 5-year survival rates of GC patients. Besides, the relationship between immune infiltration and the risk score was analyzed in the high and low risk groups. Multi Experiment Matrix (MEM) was used to analyze glycolysis-related lncRNA target genes. R “limma” package was used to analyze the mRNA expression levels of the glycolysis-related lncRNA target genes in TCGA. Gene set enrichment analysis (GSEA) was employed to further explore the biological pathways in the high-risk group and the glycolysis-related lncRNA target gene.

**Results:** A prognostic signature was conducted based on nine glycolysis-related lncRNAs, which are AL391152.1, AL590705.3, RHOXF1-AS1, CFAP61-AS1, LINC00412, AC005165.1, AC110995.1, AL355574.1 and SCAT1. The area under the ROC curve (AUC) values at 1-year, 3-year, and 5-year were 0.765, 0.828 and 0.707 in the training set, and 0.669, 740 and 0.807 in the testing set, respectively. In addition, the nomogram could efficaciously predict the 1-year, 3-year, and 5-year survival rates of the GC patients. Then, we discovered that GC patients with high-risk scores were more likely to respond to immunotherapy. GSEA revealed that the signature was mainly associated with the calcium signaling pathway, extracellular matrix (ECM) receptor interaction, and focal adhesion in high-risk group, also indicated that SBSPON is related to aminoacyl-tRNA biosynthesis, citrate cycle, fructose and mannose metabolism, pentose phosphate pathway and pyrimidine metabolism.

**Conclusion:** Our study shows that the signature can predict the prognosis of GC and may provide new insights into immunotherapeutic strategies.

## Introduction

Gastric cancer (GC) is a prevalent and deadly illness, with 1,089,103 new cases and 768,793 new deaths in 2020 [1]. There is a decrease in incidence of distal GC and an increase in incidence of proximal esophagogastric junction cancer [2]. It is reported that most of GC patients are diagnosed with advanced stage, leading to a 5-year survival rate of only 25%–30% for patients with GC in most countries [3]. The clinical treatment strategy for gastric cancer is based on surgery, chemotherapy, radiotherapy, molecular targeting of human epidermal growth factor receptor 2 monoclonal antibody and immunotherapy such as programmed cell death ligand 1 inhibitors, but the prognosis of GC patients with advanced stages remains poor. Therefore, it is necessary to establish new and effective prognostic biomarkers to improve the survival rates of GC patients.

Metabolic reprogramming, as a distinctive hallmark of malignancy [4], plays an important role in tumor diagnosis, supervision and treatment [5, 6]. One of the most common metabolic reprogramming methods is the Warburg effect, in which cancer cells rely primarily on glycolysis to generate ATP under aerobic conditions [7, 8]. This effect is associated with the tumorigenesis, invasion, metastasis, drug resistance and poor prognosis of GC [9]. Recent studies have established a mechanism for mechanically regulated glycolysis through the tripartite motif-containing protein 21 -modulated degradation of the platelet isoform of phosphofructokinase, revealing a correlation between cell metabolism and the mechanical properties of surrounding tissues [10]. In addition, the Warburg effect provides the theoretical basis for staging and recurrence assessment of clinical 18F-FDG PET/CT examination in solid cancers [11].

lncRNA is a class of noncoding RNA with more than 200 nucleotides in length, which participates in modulating chromatin function, regulating the assembly and function of different nuclear condensates, altering the stability and translation of cytoplasmic mRNAs and interfering with signaling pathways, leading to neuronal disorders, immune responses and cancer [12]. For instance, lncRNA inducing major histocompatibility complex-I and tumor immunogenicity, as a tumor immunogenic lncRNA (LIMIT), can induce the expression of major histocompatibility complex-I *via* targeting the LIMIT–GBP–HSF1 axis, promoting T-cell-mediated tumor immune response and enhancing immunotherapy efficacy [13]. Another study demonstrated that gastric cancer-associated lncRNA1 (GClnc1) could act as a scaffold lncRNA linking WDR5 and KAT2A, triggering proliferation, invasion and metastasis by activating SOD2 in GC [14]. In addition, lncRNAs, as tissue-specific and condition-specific expression molecules, are potential biomarkers and targets for cancer therapy [12, 15].

Therefore, this study aimed to explore the association between glycolysis-related lncRNA and prognosis of GC, and constructed a prognostic risk signature by analyzing gene expression data and clinical materials obtained from TCGA database.

## Materials and Methods

### Data Download

The transcriptome data and corresponding clinical characteristics of GC patients were downloaded from TCGA database (https://cancergenome.nih.gov/). Clinical data of patients with survival time less than 30 days were excluded. The clinical characteristics are showed in Table 1. 293 glycolysis-related genes were extracted from MSigDB (https://www.gsea-msigdb.org) (M5937, M11521, M5113, M27950, and M39474).

**TABLE 1:** Characteristics of patients with GC.

Clinical characteristics		Total	%
TCGA		406	100
Survival status	Survival	265	65.27
	Death	141	34.73
Age	<65 years	171	42.12
	≥65 years	232	57.14
	Unknown	3	0.74
Gender	Female	150	36.95
	Male	256	63.05
Histological grade	G1	10	2.46
	G2	149	36.70
	G3	240	59.11
	GX	7	1.72
Stage	I	56	13.79
	II	118	29.06
	III	167	41.13
	IV	39	9.61
	Unknown	26	6.40
T classification	T1	23	5.67
	T2	85	20.94
	T3	185	45.57
	T4	103	25.37
	TX	10	2.46
M classification	M0	361	88.92
	M1	27	6.65
	MX	18	4.43
N classification	N0	122	30.05
	N1	109	26.85
	N2	80	19.70
	N3	78	19.21
	NX	15	3.69
	Unknown	2	0.49

### Identification of the Prognostic Glycolysis-Related Long Noncoding RNA

Pearson correlation analysis was used to calculate the correlations between lncRNA and glycolysis-related genes. Any lncRNA with an absolute value of correlation coefficients |r| > 0.4 and *p* value < 0.01was regarded as being related to glycolysis. Univariate Cox regression analysis was carried out to identify the prognostic value of glycolysis-related lncRNA (*p* < 0.01).

### Construction of the Glycolysis-Related Long Noncoding RNA Signature

337 TCGA GC patients were randomly divided into training set (*n* = 169) and the testing set (*n* = 168) by “caret” R package. LASSO analysis was performed to prevent overfitting effects of the lncRNA using 1,000 times ten-fold cross validation. Next, the glycolysis-related lncRNA obtained from the LASSO regression were analyzed by multivariate Cox regression analysis to calculate the risk score. The risk score formula was established as follows:

$$Risk\;score=\overset{n}{\underset{i}{\sum }}coefi*Expression\;of\;xi$$

(coefi represents the coefficient and Expression of xi represents the expression value of each glycolysis-related lncRNA). GC patients were divided into low-risk and high-risk groups according to the median risk score using the formula in training set and testing set. Kaplan–Meier survival curves and log-rank test were used to analyze the overall survival (OS) in the high-risk and low-risk groups. The AUC values of the ROC curves and the concordance index (C-index) were used to evaluate the reliability of the risk score model. Besides, the relationship between prognostic associated glycolysis-related lncRNA and glycolysis-related genes was displayed by Sankey diagram *via* “ggplot” and “ggalluvial” R package.

### Nomogram Construction

A nomogram integrated clinical features (age, gender, grade and stage) and the risk score was established using the “rms” R package to assess the 1-year, 3-year and 5-year survival possibility for GC patients. Calibration curve and the C-index were applied to assess the predictive accuracy of the nomogram.

### Immunity Analysis

TIMER, CIBERSORT, CIBERSORT-ABS, QUANTISEQ, MCPCOUNTER, XCELL and EPIC algorithms were used to analyze the degree of immune cell infiltration in high-risk and low-risk groups. Heat map was used to show the types and differences of immune cells under different algorithms. In addition, single-sample gene set enrichment analysis (ssGSEA) using “gsva” (R-package) was used to further explore the difference of immune functions between the high-risk and low-risk groups. The difference in the expression of immune checkpoints between two groups was analyzed by Wilcoxon test. The immune checkpoint genes were provided in the Supplementary Table S3.

### Analysis of Glycolysis-Related Long Noncoding RNA Target Genes

MEM (https://biit.cs.ut.ee/mem/), an online database, was used to analyze glycolysis-related lncRNA target genes. R “limma” package was used to analyze the mRNA expression levels of the glycolysis-related lncRNA target genes in TCGA.

### Gene Set Enrichment Analysis

GSEA (4.1.0) was used to identify the potential pathways between the high-risk and low-risk groups. The gene set used in this study was c2.cp.kegg.v7.4.symbols.gmt, including Kyoto Encyclopedia of Genes and Genomes pathways, which were downloaded from the MSigDB. The pathways with normalized (NOM) *p*-value <0.05 and false discovery rate (FDR) < 0.05 were considered to be significantly enriched.

### Statistical Analysis

All data were analyzed in the R software (version 3.6.1). Pearson correlation analysis was used to evaluate the correlation between glycolysis-related genes and lncRNAs. Kaplan–Meier and log rank test were used to perform the relationship between clinicopathological characteristics and OS in GC patients. Chi-square test was utilized to evaluate the expression of clinicopathological manifestations of high-risk and low-risk groups. Wilcoxon test was applied to compare the difference in proportions between the risk score of the glycolysis-related lncRNA signature and the immune checkpoint. *p* value < 0.05 was considered significant.

## Results

### Construction of the Glycolysis Related Long Noncoding RNA Signature

**FIGURE 1:**
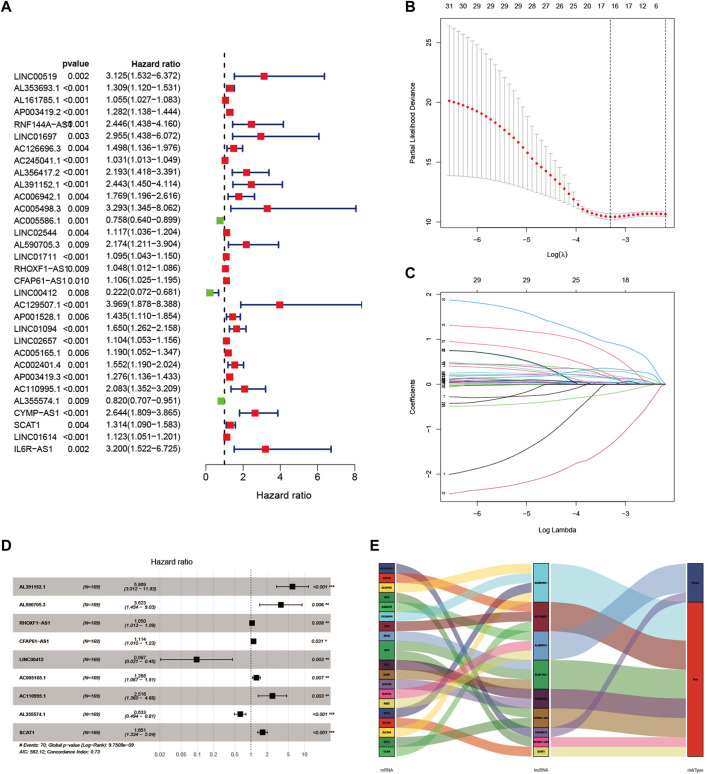
Construction of a glycolysis-related lncRNA signature in GC patients. **(A)** Univariate Cox regression analysis identified 32 lncRNAs correlated to the survival of GC patients. **(B)** The best lambda value with error bars were selected in coefficient distribution plots. **(C)** Lasso coefficient profiles of nine glycolysis related lncRNAs. **(D)** Multivariate Cox regression analysis was applied to construct the signature in the training set. **(E)** Sankey diagram of the relationship between nine glycolysis-related lncRNAs, 20 mRNAs and risk types (risk or protective).

Pearson correlation analysis was performed to explore lncRNA expression with 293 glycolysis-related genes in GC patients, and a total of 1,536 lncRNAs were considered as glycolysis-related lncRNA (Supplementary Tables S1, S2). As shown in Figure 1A, 32 lncRNAs significantly correlated to GC prognosis were identified by univariate Cox regression analysis. As shown in Figures 1B–D, we identified nine glycolysis-related lncRNA correlated with prognosis in the training set through LASSO and multiple Cox regression analysis, including AL391152.1, AL590705.3, RHOXF1-AS1, CFAP61-AS1, LINC00412, AC005165.1, AC110995.1, AL355574.1 and SCAT1. Based on the results of multivariate Cox regression analysis, a prognostic risk score model was constructed. In Figure 1E, the Sankey diagram displayed that seven lncRNAs were risk factors (AL391152.1, AL590705.3, RHOXF1-AS1, CFAP61-AS1, AC005165.1, AC110995.1 and SCAT1) and two lncRNAs were protective factors (LINC00412, AL355574.1) in GC patients. The formula as following: risk score = (1.786502 × expression of AL391152.1) + (1.287388 × expression of AL590705.3) + (0.048445 × expression of RHOXF1-AS1) + (0.107710 × expression of CFAP61-AS1) + (−2.332926 × expression of LINC00412) + (0.237407 × expression of AC005165.1) + (0.922785 × expression of AC110995.1) + (−0.457651 × expression of AL355574.1) + (0.501351 × expression of SCAT1).

### Evaluation of the Prognostic Signature in Training Set and Verification in Testing Set

**FIGURE 2:**
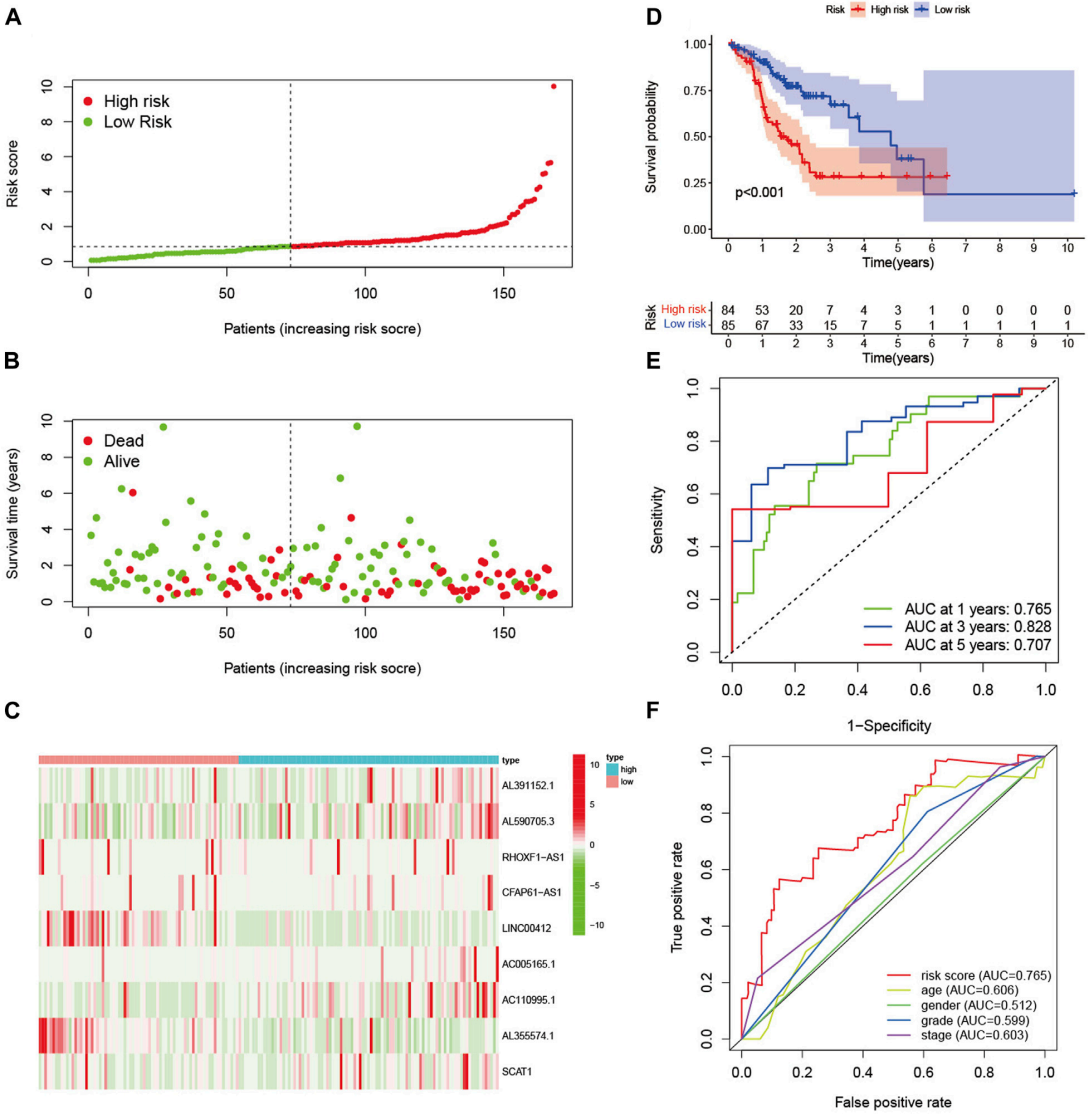
Evaluation of the signature in training set. **(A)** The distribution of risk score in the high-risk and low-risk groups. **(B)** Scatter dot plot showed the distribution of patient survival status. **(C)** Heatmap of the nine glycolysis-related lncRNAs in high-risk and low-risk groups. **(D)** Kaplan-Meier survival curves of GC patients in high-risk and low-risk groups. **(E)** ROC curves of 1-year, 3-year, and 5-year predictive signature of the training set. **(F)** The ROC curves of 1-year were constructed by risk score, age, gender, grade and stage to show the prognostic ability of each variable in training set.

**FIGURE 3:**
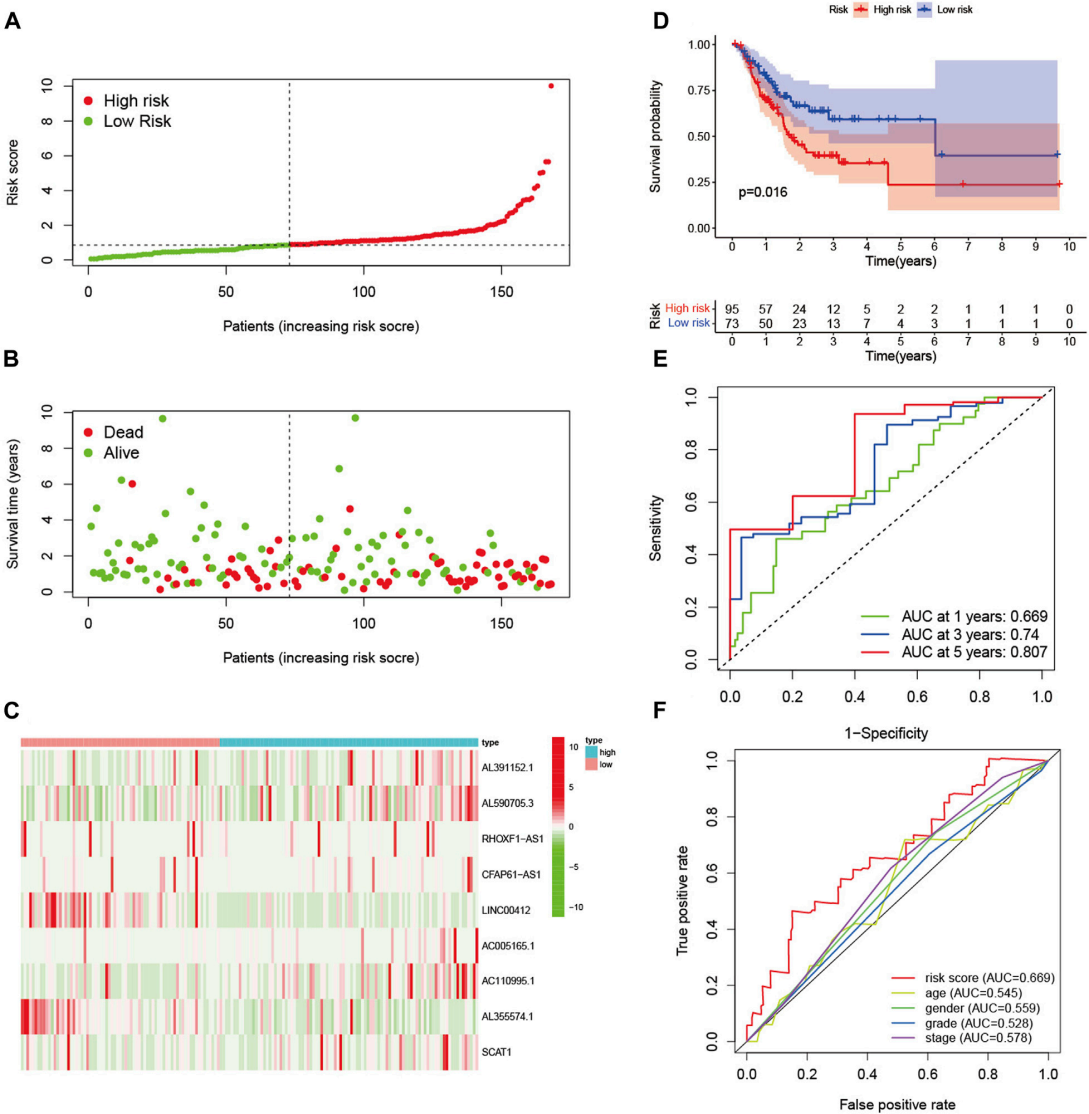
Verification of prognostic signature in the testing set. **(A)** The distribution of risk score of GC patients in the high-risk and low-risk groups. **(B)** Scatter dot plot showed the distribution of patient survival status. **(C)** Heatmap of the nine glycolysis-related lncRNAs in high-risk and low-risk groups. **(D)** Kaplan-Meier survival curves of GC patients in high-risk and low-risk groups. **(E)** ROC curves of 1-year, 3-year, and 5-year predictive signature of the training group. **(F)** The ROC curves 1-year were constructed by risk score, age, gender, grade and stage to show the prognostic ability of each variable in testing set.

In the training set, patients were divided into high-risk and low-risk groups with the median risk score as the cut-off value (Figure 2A). Figure 2B showed the distribution of risk score and survival status of GC patients. The heatmap showed the expression of nine glycolysis-related lncRNAs in the high-risk and low-risk groups (Figure 2C). Kaplan-Meier analysis indicated that the prognosis of the high-risk group was significantly worse than that of the low-risk group (*p* < 0.001) (Figure 2D). Besides, ROC curves analysis showed that the AUC of 1-year, 3-year and 5-year was 0.765, 0.828 and 0.707, respectively (Figure 2E). As displayed in Figure 2F, the risk score AUC in the training set ranked the highest among other clinical characteristics, which was 0.765. In order to evaluate the predictive efficacy of the glycolysis-related lncRNA signature, patients’ risk score was divided into low-risk (*n* = 85) and high-risk (*n* = 84) groups by using the same cut off according to the constructed formula in the training set. Similar findings were observed in the testing set. The detailed risk score, survival information, and the expression of the nine glycolysis-related lncRNAs were presented in Figures 3A–C. Kaplan-Meier analysis revealed that the survival time of gastric cancer patients in the high-risk group was lower than that of patients in the low-risk group in the testing set (log-rank *p* = 0.016) (Figure 3D). As shown in Figure 3E, the AUC values of 1- year, 3- years, and 5-years ROC curves were 0.669, 0.740, and 0.807, respectively. The AUC of the nine glycolysis-related lncRNAs in the testing set predicting overall survival reached 0.669, which was the leading variable compared to other factors (Figure 3F).

### Establishment of a Nomogram for Prognostic Prediction in Gastric Cancer Patients

**FIGURE 4:**
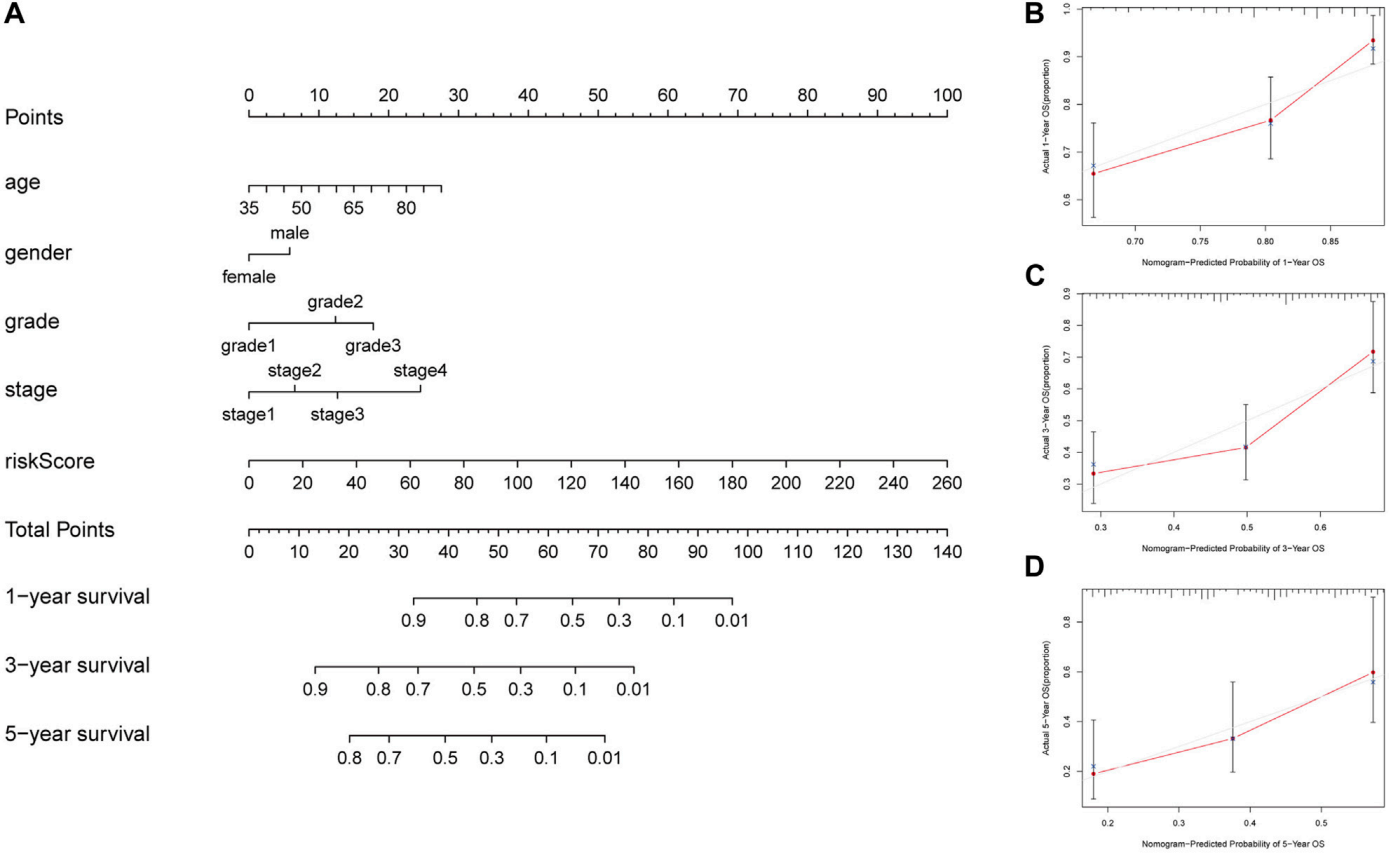
Construction of a nomogram to predict prognosis in patients with GC at 1-year, 3-year, and 5-year. **(A)** Nomogram to predict the OS at 1-year, 3-year, and 5-year based on age, gender, grade, stage and risk score **(B)** The calibration curve of the nomogram at 1-year. **(C)** The calibration curve of the nomogram at 3-year. **(D)** The calibration curve of the nomogram at 5-year.

A nomogram containing age, gender, grade, stage and risk score was constructed to predict the 1-year, 3-year, and 5-year OS rates of GC patients in TCGA, which displayed that the higher the total score, the shorter the survival time (Figure 4A). As shown in Figures 4B–D, the calibration curves of the nomogram for the survival probability at 1- year, 3-year, and 5-year demonstrated that the predicted survival rates were approximately equal to the actual survival rates. The C-index values of the nomogram in TCGA was 0.673, which suggested a promising clinical application value in predicting the long-term survival probability of GC patients.

### Analysis of the Risk Score and Clinicopathologic Features of Gastric Cancer Patients

**FIGURE 5:**
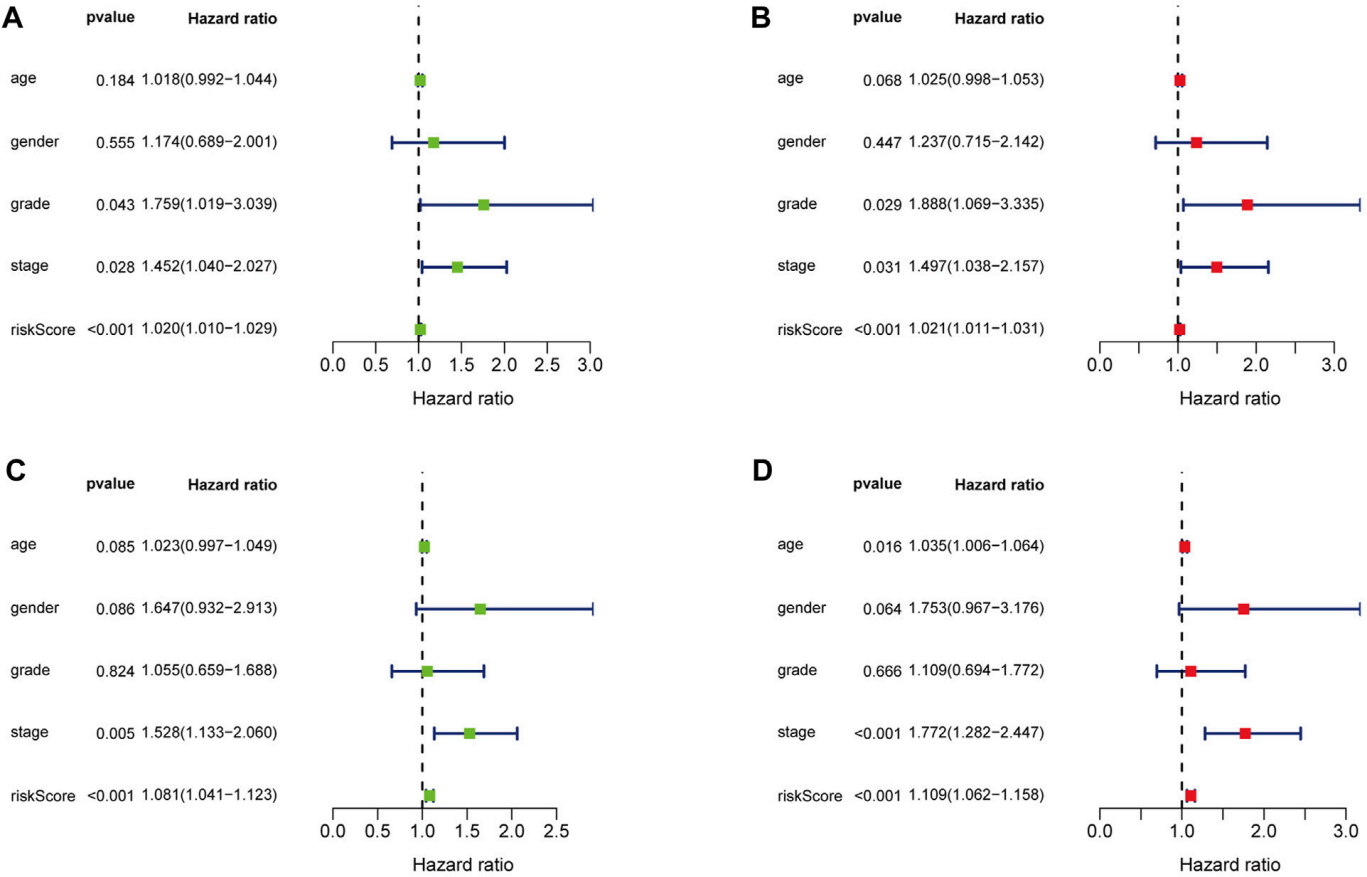
Cox regression analysis of clinical characteristics related to OS in training set and testing set. **(A)** Univariate Cox regression analysis showed that grade, stage and the risk score related to OS in training set. **(B)** Multivariate Cox regression analysis showed that grade, stage and the risk score were independent prognostic factors in training set. **(C)** Univariate Cox regression analysis showed that stage and the risk score related to OS in testing set. **(D)** Multivariate Cox regression analysis showed that age, stage and the risk score were independent prognostic factors in testing set.

**FIGURE 6:**
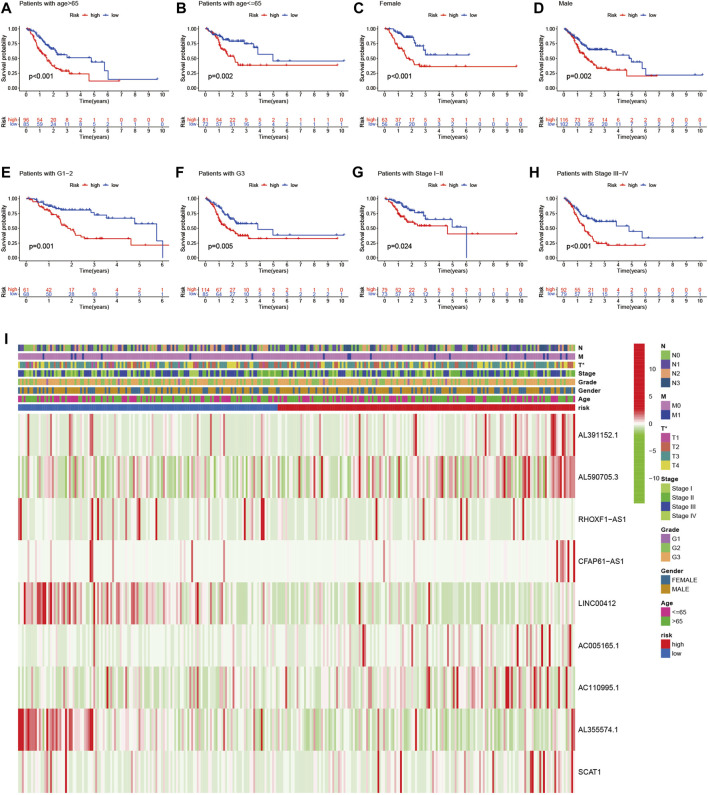
Prognostic significance and subgroup analysis of the glycolysis-related lncRNA signature. **(A,B)** age; **(C,D)** gender; **(E,F)** grade; **(G,H)** stage. **(I)** Heatmap of the glycolysis-related lncRNAs expression in the signature and the clinicopathological features of patients with GC (*p < 0.05).

Univariate and multivariate Cox regression analysis revealed that the risk score of the signature can serve as an independent factor for the prognosis of GC patients in both the training set and the testing set (Figures 5A–D). To further assess the prognostic value of the signature in GC patients, we categorized age, gender, grade and stage into high-risk and low-risk groups based on the median risk score. Kaplan-Meier analysis showed that the OS of GC patients in the high-risk group was worse than that in the low-risk group (*p* < 0.05) (Figures 6A–H). Then a heatmap was used to depict the relationship between lncRNA expression, the risk score and clinicopathological characteristics including age, gender, grade, stage and TNM status. The results revealed that there was a significant difference between the high-risk group and the low-risk group in T stage (*p* < 0.05) (Figure 6I). Consequently, these results suggest that glycolytic lncRNA signaling can be used as a method for prognostic assessment of clinicopathological factors in GC patients.

### Analysis of Immune Status in the High-Risk and Low-Risk Group

**FIGURE 7:**
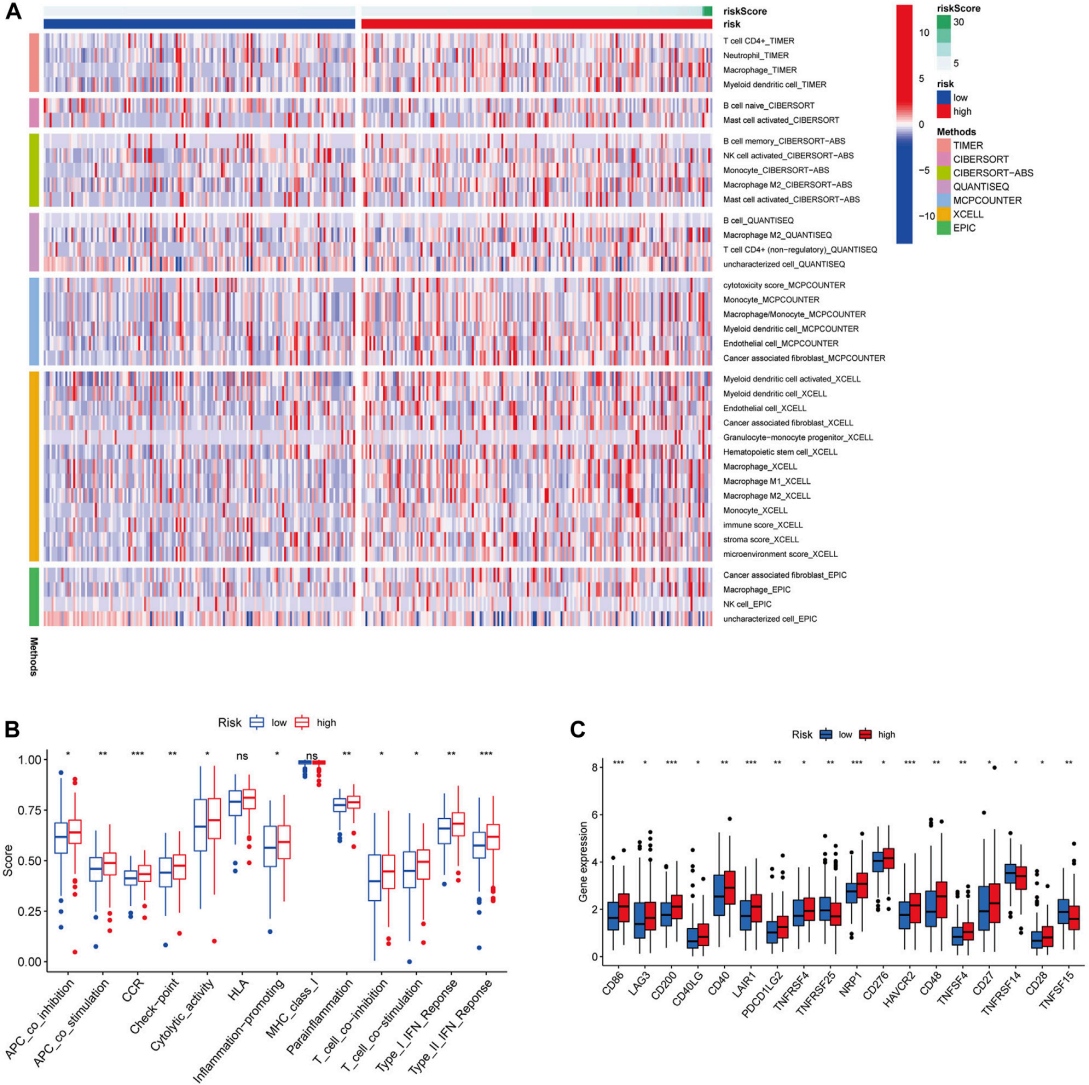
Different immune cells statuses in low-risk and high-risk groups. **(A)** Distribution of immune cells in low-risk and high-risk groups. **(B)** Differences in immune function between low-risk and high-risk groups. **(C)** Expression levels of immune checkpoints. (*p < 0.05) (**p < 0.01) (***p < 0.001).

To explore the relationship between immune cell infiltration level and risk score in the TCGA dataset, we generated a heatmap with TIMER, CIBERSORT, CIBERSORT-ABS, QUANTISEQ, MCPCOUNTER, XCELL, EPIC algorithms. The heatmap showed that there were significant differences in the risk scores of immune cells in the two groups, with higher immune cells in the high-risk group than in the low-risk group (Figure 7A). ssGSEA analysis showed that APC co-inhibition, APC co-stimulation, chemokine receptors, checkpoint, cytolytic activity, inflammation promoting, para-inflammation, T cell co-stimulation, T cell co-inhibition, type I INF response and type II INF response were higher in the high-risk group than in the low-risk group (Figure 7B). In addition, the expression levels of immune checkpoints including CD86, LAG3, CD200, CD40LG, CD40, LAIR1, PDCD1LG2, TNFRSF4, NRP1, CD276, HAVCR2, CD48, TNFSF4, CD27 and CD28 were relatively higher in the high-risk group compared with the low-risk group (Figure 7C). In contrast, TNFRSF25, TNFRSF14 and TNFSF15 were higher in the low-risk group. Above all, we can summarize that the risk score of glycolysis-related lncRNA signature might have certain application in immunotherapy of GC patients.

### Gene Set Enrichment Analysis of the Glycolysis-Related Long Noncoding RNA Signature

**FIGURE 8:**
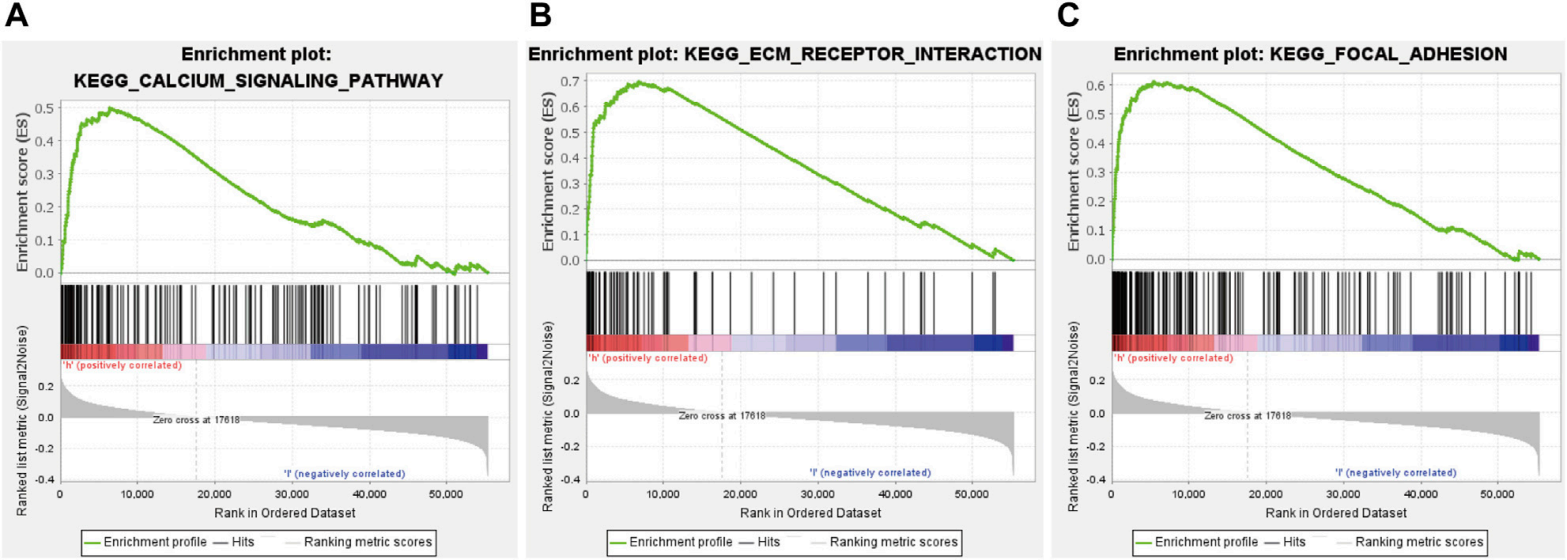
significant signaling pathways identified by GSEA. **(A)** calcium signaling pathway. **(B)** ECM receptor interaction. **(C)** focal adhesion.

GSEA was conducted to explore the underlying molecular mechanism of glycolysis-related lncRNA signature in high-risk and low-risk groups. The results revealed that the signature based on glycolysis-related lncRNA in the high-risk group was mainly involved in calcium signaling pathway, ECM receptor interaction and focal adhesion (Figures 8A–C). However, there was no significant pathway enrichment in low-risk group. Taken together, these results suggested that these pathways may influence the prognosis of GC patients.

### Analysis of Glycolysis-Related Long Noncoding RNA Target Genes

**FIGURE 9:**
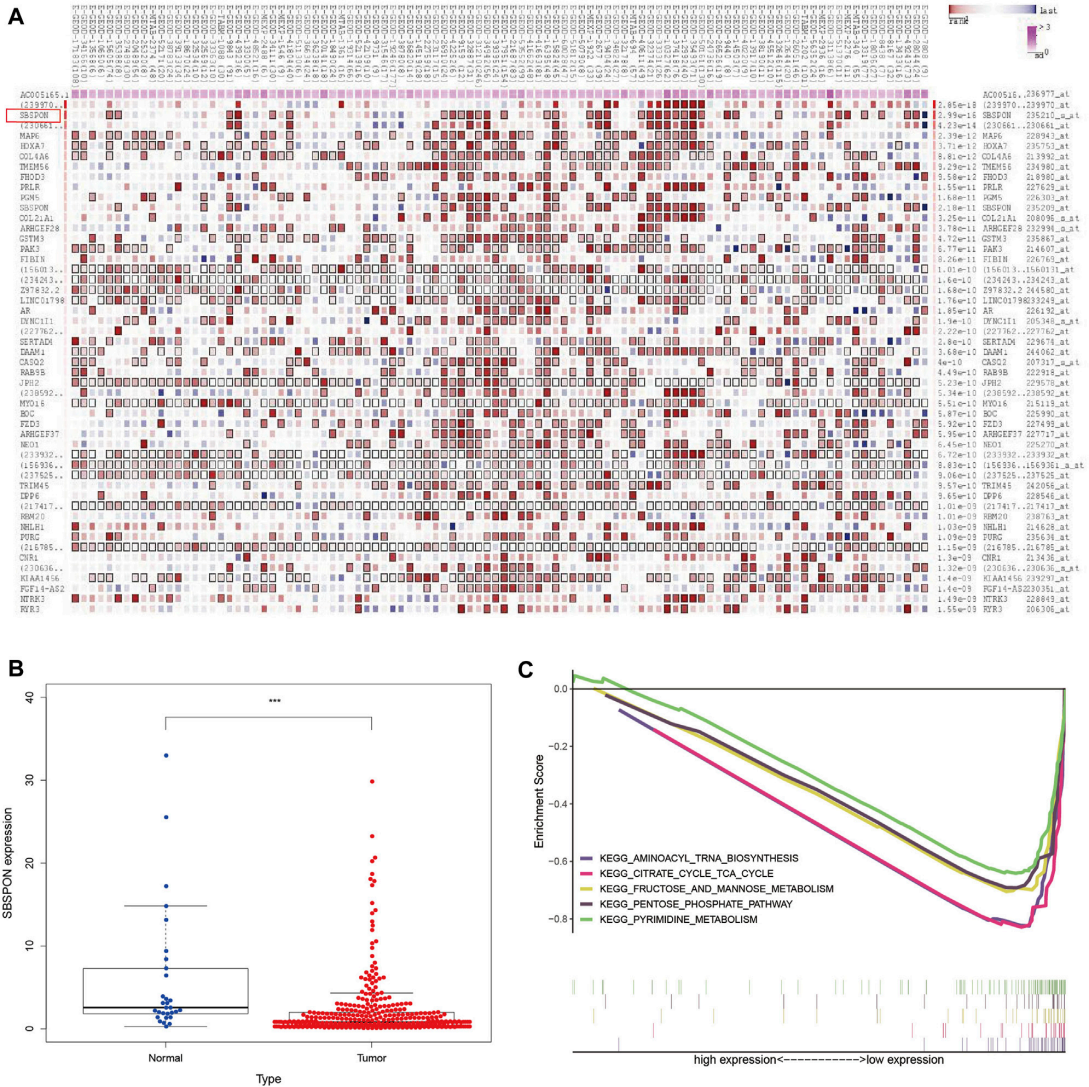
Analysis of glycolysis-related lncRNA target genes. **(A)** SBSPON is the most likely target gene of AC005165.1 by MEM. **(B)** SBSPON is lowly expressed in gastric cancer tissues (***p < 0.001). **(C)** GSEA suggests that SBSPON is involved in aminoacyl-tRNA biosynthesis, citrate cycle, fructose and mannose metabolism, pentose phosphate pathway and pyrimidine metabolism.

In order to find the target genes of the nine lncRNAs, we analyzed the target genes of these nine lncRNAs by MEM. Among the nine lncRNAs, only AC005165.1 had a probe name, which could be further analyzed. In MEM, SBSPON was identified as the gene with the highest score by target gene analysis of AC005165.1, the results of which are added in Figure 9A. We further analyzed the mRNA level expression of SBSPON in TCGA and found that it was lowly expressed in gastric cancer tissues (Figure 9B). There are few studies on SBSPON, it is predicted that SBSPON is a structural component of extracellular matrix and colocalizes with collagen-containing extracellular matrix. GSEA indicates that the low expression of SBSPON in gastric cancer tissues is related to aminoacyl-tRNA biosynthesis, citrate cycle, fructose and mannose metabolism, pentose phosphate pathway and pyrimidine metabolism (Figure 9C).

## Discussion

Many methods were used to predict the survival rate of GC patients. It has been reported that GC patients with high expression levels of TP and/or growth arrest and DNA damage-inducible A (GADD45A) have a significantly lower survival after neoadjuvant chemotherapy [16]. In GC, intrinsic subtypes can predict patient survival, and the OS of patients with genomic intestinal tumor is better than that of patients with genomic diffuse tumor [17]. Recently, a noninvasive imaging signature for immune score of GC based on IHC staining of surgical specimens by radiomic analysis of pretreatment CT images effectively assesses recurrence and prognostic significance in gastric cancer [18, 19]. However, the survival prediction of GC is still dismal, the survival period for patients with GC ranges from less than 5 months to over 10 years [20]. Accumulated evidence showed that lncRNA was reported to be associated with the outcome of GC. For instance, lncRNA insulin growth factor 2 antisense (IGF2-AS) was significantly higher in GC tissues compared with normal tissues and correlated with poor survival, and the upregulation of IGF2-AS expression can significantly promote cell viability, migration and invasion of GC cells by miR-503/SHOX2 axis [21].

Based on TCGA database, we identified nine glycolysis-related prognostic lncRNA (AL391152.1, AL590705.3, RHOXF1-AS1, CFAP61-AS1, LINC00412, AC005165.1, AC110995.1, AL355574. d SCAT1) by univariate Cox regression analyses, LASSO regression analysis and multivariate Cox regression analyses, and then relied on it to construct a prognostic risk score signature to predict the OS of the patients with GC. The results showed that the OS of GC patients in the high-risk score group was shorter than that of the low-risk score group. Kaplan–Meier survival curves and ROC curves verified the high prognostic value of the risk score of our prognostic signature in training set and testing set. Furthermore, the nomogram diagram, including age, gender, grade, stage and risk score, showed a high accuracy in the 1-year, 3-year and 5-year OS outcomes in individual GC patient. In addition, the results of univariate and multivariate Cox regression analysis showed that the glycolysis-related prognostic lncRNA signature consisting of nine screened lncRNAs were independent of other clinicopathological parameters and could be used to predict OS in gastric cancer patients. The prognostic analysis was equally significant in the subgroups based on different pathological types. The glycolysis-related prognostic lncRNA signature was also able to identify significant differences in therapeutic indicator and immunotherapy responses in GC patients. MEM suggested that SBSPON is the target gene analysis of AC005165.1.GSEA results indicated that calcium signaling pathway, ECM receptor interaction, and focal adhesion were significantly enriched in the high-risk group, which provided a more reasonable and convincing explanation for the poor prognosis of the high-risk group. Furthermore, GSEA also showed that SBSPON was involved in aminoacyl-tRNA biosynthesis, citrate cycle, fructose and mannose metabolism, pentose phosphate pathway and pyrimidine metabolism.

Cancer progression to the metastatic stage is still uncontrolled, and the treatment outcomes with surgery, radiation, chemotherapy and molecularly targeted agents remain largely unsatisfactory, underscoring the need to develop new therapies [22, 23]. Immunotherapy is an anti-tumor approach that kills and eliminates tumor cells by stimulating the host immune system and has become one of the most important and successful cancer treatment categories [24, 25]. Currently, the most promising approach in cancer immunotherapy to activate therapeutic antitumor immunity is immune checkpoint blocking [26]. Cancer immunotherapy targeting immune checkpoint blockade has been shown to significantly improve the prognosis of patients with malignant tumors compared to conventional treatment [27–29]. Cellular metabolism plays an important role in cancer cell proliferation, drug resistance and invasion, as well as functional activation of immune cells [30, 31]. In the field of immunology, lncRNA has been shown to play a positive role in innate immune response and T cell development, differentiation, and activation by regulating protein-protein interactions or *via* the ability to interact with RNA and DNA base pairs [32]. In our study, after comparing the difference of tumor infiltrating immune cells between high-risk and low-risk groups, patients who had tumor with a high-risk score had high levels of immune cell infiltration, which might be a potential target for cancer treatment. KEGG pathway enrichment analysis of GSEA demonstrated that calcium signaling pathway, ECM receptor interaction, and focal adhesion may be important factors leading to poor OS outcome in high-risk group patients. Calcium signaling can regulate multiple aspects of immune cell biology, including differentiation, effector function, and gene transcription [33–35]. In addition, Ca^2+^ dependent signaling is mainly involved in angiogenesis, immune evasion, metastasis, and drug resistance in cancer [36]. ECM receptor interaction participates in regulating cell adhesion, motility and cell signaling, affecting cell functions and differentiation and so on [37]. For example, integrins with p53 as classical ECM receptors could regulate apoptosis of cells after DNA damage [38]. Focal adhesion is also a classic pathway affecting cell migration. Kevin et al. found that CD155/PVR reduced substrate adhesion, cell spreading, focal adhesion density and the number of actin stress fibers in a substrate-dependent manner, even affected the progression of glioma cells [39]. It’s reported that SCAT1 as a component of the three-lncRNA signature predicting pathological response and outcome in esophageal squamous cell carcinoma with neoadjuvant chemoradiotherapy shows a compellent prognostic value in patients [40].

Unfortunately, although there were no reports focusing on the eight lncRNAs, sankey diagram indicates that the glycolysis-related lncRNA may regulate glycolytic function either directly or indirectly. Because all the data were obtained from public database, and the sample size was relatively small, further a multicenter, large-sample clinical study is needed to verify the prognostic significance of the glycolysis-related lncRNA signature in GC. In conclusion, we identified and verified a prognostic model based on glycolysis-related lncRNA to evaluate the prognosis of GC patients, and predict whether GC patients are suitable for immunotherapy. Therefore, we expect the signature could be applied as a potential prognostic indicator in clinical treatment and provide a new sight in immunotherapy.

## Data Availability

The datasets analyzed in this study can be found in online repositories. The names of the repository/repositories and accession number(s) can be found in the article/Supplementary Material.

## Supplementary Material

Supplementary Table S1 293 glycolysis-related genes.

Supplementary Table S2 1536 glycolysis-related lncRNAs.

Supplementary Table S3 47 genes related to immune checkpoints.
